# A Bioengineered Quercetin-Loaded 3D Bio-Polymeric Graft for Tissue Regeneration and Repair

**DOI:** 10.3390/biomedicines10123157

**Published:** 2022-12-07

**Authors:** Archna Dhasmana, Sumira Malik, Anuj Ranjan, Abhishek Chauhan, Hanaa M. Tashkandi, Shafiul Haque, Rajaa Al-Raddadi, Steve Harakeh, Gökhan Zengin

**Affiliations:** 1 Himalayan School of Biosciences, Swami Rama Himalayan, Jolly Grant, Dehradun 248140, Uttarakhand, India; 2 Amity Institute of Biotechnology, Amity University Jharkhand, Ranchi 834001, Jharkhand, India; 3 Academy of Biology and Biotechnology, Southern Federal University, 344006 Rostov-on-Don, Russia; 4 Amity Institute of Environmental Toxicology, Safety and Management, Amity University, Noida 201303, Uttar Pradesh, India; 5 Department of General Surgery, Faculty of Medicine, King Abdulaziz University, Jeddah 21589, Saudi Arabia; 6 Research and Scientific Studies Unit, College of Nursing and Allied Health Sciences, Jazan University, Jazan 45142, Saudi Arabia; 7 Community Medicine Department, Faculty of Medicine, King Abdulaziz University, Jeddah 21589, Saudi Arabia; 8 King Fahd Medical Research Center, and Yousef Abdullatif Jameel Chair of Prophetic Medicine Application, Faculty of Medicine, King Abdulaziz University, Jeddah 21589, Saudi Arabia; 9 Department of Biology, Science Faculty, Selcuk University, Konya 42250, Turkey

**Keywords:** herbal, quercetin, drug, graft, biocompatibility, regeneration

## Abstract

Phytochemicals extracted from plant sources have potential remedial effects to cure a broad range of acute to severe illnesses and ailments. Quercetin is a flavonoid isolated from different dietary sources such as vegetables and fruits, exhibiting strong anti-inflammatory, anti-oxidative and non-toxic effects on the biological system. However, the direct uptake or administration of quercetin results in loss of functionality, poor activity, and reduced shelf-life of the bioactive component. In this regard, to improve the uptake, potential, and efficiency of natural components with prolonged storage in the host’s body after administration, numerous polymer drug delivery systems have been created. In the current study, three-dimensional (3D) porous (porosity: 92%; pore size: 81 µm) bio-polymeric foaming gelatin–alginate (GA) beads were fabricated for the entrapment of quercetin as therapeutic drug molecules—gelatin–alginate–quercetin (GAQ). The GAQ beads showed a significant uptake of quercetin molecules resulting in a reduction of reduced porosity up to 64% and pore size 63 µm with a controlled release profile in the PBS medium, showing ~80% release within 24 h. Subsequently, the GAQ beads showed remarkable antioxidant effects, and 95% anti-inflammatory activities along with remarkable in vitro cell culture growth and the observed proliferation of seeded fibroblast cells. Thus, we can conclude that the consistent release of quercetin showed non-toxic effects on normal cell lines and the bioactive surface of the GAQ beads enhances cell adhesion, proliferation, and differentiation more effectively than control GA polymeric beads and tissue culture plates (TCP). In summary, these findings show that these GAQ beads act as a biocompatible 3D construct with enormous potential in medicinal administration and tissue regeneration for accelerated healing.

## 1. Introduction

Natural components, including biopolymers and bioactive compounds isolated from different natural resources, such as plants, animals, and microbes, have been widely used for biomedical applications [1,2]. A wide range of bioactive molecules and phytochemicals have been used to treat several types of diseases such as diabetes, chronic inflammation, skin infections, ulcers, metabolic deformities and specifically targeting abnormal cell growth in cancer. Quercetin is a natural polyphenolic component or flavonoid classified as a phytoestrogen of biochemical origin. It is also known as vitamin P because of its wide availability in primary natural dietary sources, such as red onion, red grapes, tea, green leafy vegetables, apples, and citrus fruits [3]. A potent flavonoid inhibits the activity of several enzymes such as acetylcholinesterase and cyclo-oxygenase. Moreover, these flavonoids possess antioxidant, anti-inflammatory, anticarcinogenic, anti-thrombin, anti-hypertensive, antiviral, and antibacterial activity [4,5]. Therefore, quercetin has been widely used as a therapeutic drug molecule to treat cancer, heart disease, and UV-induced damage [6,7].

The poor solubility and degradation of the Quercetin molecule during the local administration inside the host body reduce its therapeutic potential and efficiency [8]. The drug delivery system, i.e., biocompatible, biodegradable, controlled drug release, showed the prolonged effect of the drug with maximum therapeutic index and reduced the side-effects [9,10]. 3D matrices fabricated from polymers and composites are widely used as a model to study drug release profiles, in vivo tissue–drug interactions, and targeted drug delivery systems [11,12]. Hence, quercetin is efficiently loaded in the different polymeric systems and studied for its effect on the therapeutic index [13,14,15].

Different polymers have been studied and used as carriers for quercetin to enhance its therapeutic activity. Micro-emulsions of quercetin were prepared for the local administration of the drug molecule to study their effects under in vitro and in vivo animal systems to prevent UV-induced skin damage [16,17]. Fahlman and Krol reported that the plants and animal tissue treated with quercetin resist UV-induced mutations, and after the UV treatment, the Quercetin molecules slowly decompose into the non-toxic products [18]. Dias et al. fabricated quercetin-impregnated N-carobxylbutyl chitosan and agarose films for sustained release profiles and faster wound healing [19]. Natarajan et al. synthesized quercetin-containing polycaprolactone (PCL)–gelatin microspheres, coated with collagen to enhance the bio-adhesion of the microsphere and to prolong control drug release [20]. Another polymeric drug delivery system, polyethylene glycol (PEG)-12-hydroxy stearate, also improved the solubilization of quercetin up to five times [21]. Several studies reported that nanoemulsion as a drug delivery system increased the bio-accessibility of drugs. Pool et al. also reported that quercetin in nanoemulsions showed better bio-accessibility when compared to crystalline quercetin [22]. Wang et al., 2016, fabricated quercetin-loaded nanoliposomes (QUE-NLs) to study apoptotic cell death of cancerous tissue at a low concentration of 100 μM [16,23]. The polyvinylpyrrolidone (PVP)–quercetin composite microparticles were fabricated by coaxial electrospraying methods to enhance the solubility and infusion properties for oral or sublingual administration [24]. Polylactic acid (PLA) and polylactic co-glycolic acid (PLGA) are biodegradable and biocompatible polymers used to encapsulate and deliver the bioactive molecule Quercetin. Polymer-encapsulated Quercetin molecules strongly induced apoptosis of cancer cells [25].

Physical entrapment of bioactive molecules or drugs into the biopolymeric matrices provides a biocompatible, bio-accessible, controlled drug-delivery system. The loading or entrapment of biomolecules in polymeric matrices is usually done by mixing, immersion, and physical adsorption [26]. Three-dimensional (3D) polymeric foam scaffolds are the simplest and cheapest extracellular matrix (ECM)–biomimetic matrix used for tissue engineering studies [27]. The various natural high foam-generating polymers, such as alginate, gelatin, and silk, were used to form foam scaffolds, with or without surfactant [28].

Alginate is a polysaccharide isolated from seaweed, commonly used as a biopolymer, and has a more comprehensive application. Alginate has good mechanical and foam stability but poor surface functionality for cellular adherence, which limits its applicability [29]. In recent studies, different types of polymeric blends and composites of alginate with other polymers and non-polymeric components were designed to overcome the abovementioned limitations of alginate [30,31].

Correspondingly, gelatin is hydrolyzed from collagen protein, having a short peptide Arg–Gly–Asp (arginine–glycine–aspartate) sequence, which helps in cell attachment and proliferation [32]. It is biocompatible, biodegradable, and one of the most potent clinically approved biomaterials. In earlier studies, to overcome the limitation of both natural polymers, alginate and gelatin blends were used to fabricate polymeric matrices or scaffolds, having significant biocompatibility due to the presence of gelatin short peptides and improving biomechanical strength of alginate [33,34,35].

Therefore, gelatin and alginate are used as a polymeric matrix for the entrapment of drugs and bioactive components to improve their shelf life and enhance activity and efficiency under in situ conditions [33,36,37]. Pindolol-loaded alginate–gelatin polymeric cross-linked beads fabricated by solvent-free techniques showed the controlled release of pindolol with improved retention within the bead’s matrix [38]. Subsequently, alginate–gelatin microsphere beads were designed for the controlled release of endosulfan [39].

In this study, we fabricated a Quercetin-loaded biopolymeric foam scaffold to protect the therapeutic compound during passage through the body as a carrier and mediator/activator of controlled release. Here, we used alginate–gelatin foaming used for the entrapment of the quercetin as a polysaccharide–protein porous matrix for the drug-delivery system and a tissue-engineered scaffold for better cell signaling. Subsequently, the combination of polysaccharide and protein molecules enhances the drug encapsulation and release efficiency. However, quercetin, as a bio-active molecule, protects the healthier cell from oxidative damage and enhances cell growth, and the synergistic effect of polymeric components with quercetin could enhance the biocompatibility and anti-inflammatory and anti-oxidative effects.

## 2. Materials and Methods

### 2.1. Materials

Alginate (300 kDa), gelatin (bovine skin), phosphate-buffered saline (PBS), quercetin, calcium chloride, and acetic acid were obtained from Hi-Media, India Limited. The samples were prepared and characterized in PBS and double-distilled water.

### 2.2. Quercetin-Loaded Polymer Scaffold

**Figure 1:**
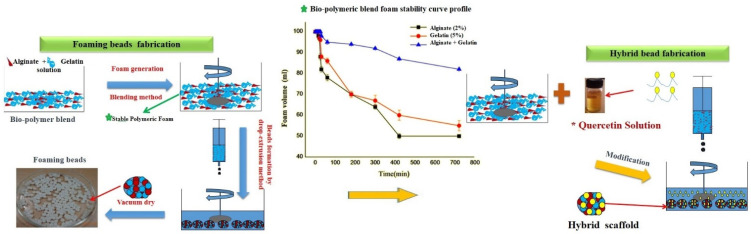
Schematic presentation of biogenic 3D polymeric foaming bead fabrication and quercetin-loaded hybrid beads with polymeric foaming stability curve showing significant foaming stability with time. Here, Green * labelling represents the foam stability curve of the polymeric blend indicates the stable foam generation of alginate-gelatin polymeric blend to bead fabrication and red * for quercetine solution for the modified alginate-gelatin bead formation.

Natural polymeric gelatin and alginate polymeric foaming scaffolds were used for fabrication. 3D porous bio-polymeric beads were fabricated simply by foaming generation and crosslinking the same in the CaCl_2_ solution by the drop-extrusion method. A stable foam of alginate and gelatin polymeric blend was generated by blending at high speed in the mechanical stirrer and subsequently cross-linking in the CaCl_2_ solution by dropping through the syringe (Figure 1). Prepared beads were vacuum-dried and used as gelatin–alginate (GA) controlled bead samples. Further to fabricating bio-polymeric blend-modified beads i.e., gelatin–alginate–quercetin (GAQ), quercetin was used as a drug molecule to encapsulate in different concentrations (Table 1). Briefly, the generated polymeric foam was drop-wise added into a solution containing different concentrations of quercetin solution and CaCl_2_ in the ratio of 1:10. After 10 h incubation, the GAQ bead properly cross-linked with the quercetin molecule was exposed to a vacuum for 12 h to form spongy or porous structures. The macroscopic size of the freshly prepared beads was measured by a Vernier caliper.

### 2.3. Ultrastructure and Chemical Linkage Analysis

The ultrastructure drug-loaded gelatin–alginate beads (GAQ) and control beads (GA) were examined under an FESEM (field emission scanning electron microscope) by using standard protocols [40]. The drug molecules loaded onto the bead’s surface, as well as the average porosity and pore size, were examined in the captured images and analyzed using ImageJ software. However, the FTIR (Fourier transform infrared spectroscopy) analysis of beads samples was performed for the semi-quantitative functional analysis and identification of intermolecular interactions between the different compounds. The FTIR spectral peaks obtained from the pure drug, pure polymer solution, polymer blend, and drug-loaded beads were analyzed for proper entrapment of drug molecules on the surface of the polymeric bead.

### 2.4. Determination of Drug Encapsulation and Release Efficiency

Each sample of 50 mg beads was weighed, crushed, and dispersed in 100 mL of 1XPBS at a neutral pH. Dissolved samples were kept on a shaker at 200 rpm for 2 h. After complete dispersions, samples were filtered and examined at 266 nm by a UV spectrophotometer. The following formula determines the encapsulation efficiency.

Encapsulation efficiency = (Aq/Tq) × 100

where Aq is the drug content of treated beads and Tq is the total drug present in beads.

Further, the total immersion method was used to study the release of the quercetin profile from the surface of the gelatin–alginate bead. Each sample absorbance was recorded using a UV-Vis spectrophotometer, and the calibration curve of quercetin (50 µg/mL) was prepared for monitoring the drug release profile. The GAQ beads were immersed in 150 mL of 1X PBS and placed in a shaking water bath at 37 °C. After 12 h of incubation, 2 mL of the sample was taken out at a regular time interval of 30 min, and the absorbance was taken at 412 nm. After measuring the absorbance, the withdrawal sample was reimbursed into the main flask to make a final constant volume. The drug release percentage was measured by comparing it with the standard calibration curve until complete release occurred.

### 2.5. In Vitro Anti-Oxidative and Anti-Inflammatory Response

The antioxidant activity of the fabricated polymeric beads was measured in vitro by the 2, 2-diphenyl-1-picrylhydrazyl (DPPH) assay method as described in the protocol [41]. Briefly, prepared reagents were added in test tubes sequentially: blank—3.8 mL of ethanol; control—3.5 mL of ethanol and 300 µL DPPH assay; test sample—500 µL of the sample, 3 mL of absolute ethanol and 300 µL of DPPH assay. Subsequently, all the samples were incubated in the dark for 30 min at room temperature and absorbance (Abs) was measured at 517 nm to calculate antioxidant activity by the formula given below:

$$\mathrm{AA}\%=100-\left[\frac{Abs_{sample}-Abs_{control}}{Abs_{control}}\right]\times 100$$

Afterwards, the anti-inflammatory activity of the biogenic beads was estimated by the human red blood cell (HRBC) membrane sterilization method [42,43]. In brief, blood samples of healthy donors were mixed in equal rations (1:1) and sterilized with Alsever’s solution. The testing samples were mixed in different concentrations separately in 1 ml PBS buffer, 2 mL hyposaline, and 0.5 mL HRBC suspension, followed by incubation at 37 °C for 30 min. After incubation, samples were centrifuged for 10 min at 5000 rpm and checked for their absorbance at 560 nm. Here, we estimated the amount of hemoglobin content and the hemolysis percentage by taking the negative control group as 100% and calculating as given in the formula given below:

$$\mathrm{HRBC}\;\mathrm{membrane}\;\mathrm{protection}\;=100-\left(\frac{\mathrm{Abs}\;\mathrm{of}\;\mathrm{test}\;\mathrm{sample}}{\;\mathrm{absorbance}\;\mathrm{of}\;\mathrm{control}}\right)\;100$$

### 2.6. In Vitro Cell Culture

L929 mouse fibroblast cells were used to determine the cytotoxic effect and the effect of the fabricated beads on cell proliferation by the SEM image analysis and MTT cell growth assay analysis [40]. In brief, to perform the cell culture study, all the prepared dried bead samples, GA and GAQ, were soaked overnight into the 48-well tissue culture plate (TCP) containing DMEM culture medium at 37 °C in a CO_2_ incubator to check the sterility before cell seeding. Incubated beads have a clear medium without any turbidity, indicating no contamination, and were selected for the next step of cell seeding. Subsequently, 10 µL of cell suspension (~1 × 10^3^ cells) was seeded over the incubated beads matrix evenly and fresh cell culture nutrient medium was added to the well. Here, the TCP well containing only cells without any beads was used as a negative control, with GA beads as the positive control and GAQ beads of different concentrations as test samples. Further, the TCP-containing cells seeded with or without beads were incubated at 37 °C in a CO_2_ incubator and we measured the cell growth and viability by performing an MTT assay and checking the absorbance at a wavelength of 490 nm at the regulative time periods of 3, 7 and 14 incubation day by using a microplate reader. However, the cell growth and neo-ECM formation for the tissue regeneration were measured by histological analysis of the microscopic samples of the captured FESEM images by ImageJ software.

## 3. Results & Discussion

### 3.1. Physicochemical Analysis of Drug Scaffold

Long standing foam constancy of the polymeric sample is an essential aspect of the intact 3D porous structure, which is simply formed by self-crosslinking of monomeric submits and generates stable foam. Alginate has suitable viscosity and foam stability due to its amphiphilic nature [29]. However, in the case of gelatin (5 wt%), the foam was stable even after 40 min. A polymeric blend of 2% alginate and 5% gelatin produced a large amount of foam and a highly stable foam for more than 30 min without any surfactant. The opposite charge of amino and carboxylate groups in alginate was supposed to interact with gelatin and generate stable foam. In earlier studies, the researcher reported that 5% gelatin and 2% alginate show highly stable foam generation [44].

Subsequently, quercetin-encapsulated foaming beads of average bead size 1.960 ± 46 mm were successfully fabricated from the natural polymeric gelatin–alginate without using surfactant (Table 1, Figure 1). The interconnected polymeric 3D ultrastructure between alginate, gelatin, and calcium chloride was established due to cross-linkage for the entrapment of the quercetin drug molecule in the matrix. It was reported that quercetin also has a cross-linking property and is widely used for the cross-linking of polymers to enhance the stability of the polymer [45,46,47].

**Figure 2:**
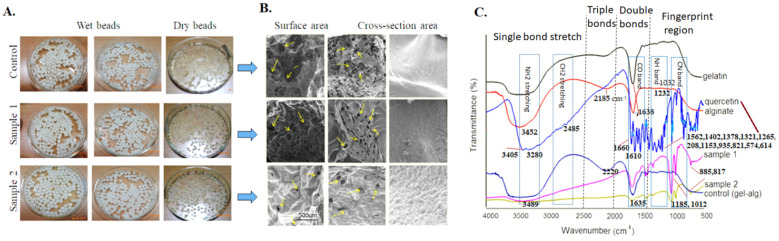
The ultrastructure of polymeric beads- (**A**) Macroscopic examination (by Vernier caliper in millimeters), (**B**) microscopic FESEM images at 1 kX (scale bar 500 µm), and (**C**) FTIR spectra of all the controlled and modified beads samples, respectively.

The highly porous structure facilitates the cell invasion and diffusion of cells and nutrients for better tissue regeneration. FESEM images of fabricated beads at a higher magnification showed the interconnected pores with good porosity up to 98% (mean porosity 88.56 ± 2.03%) for significant cellular growth (Figure 2a, Table 2). In an earlier study, a foaming scaffold fabricated simply by the gas-foaming method showed porosity ranges from 87 to 97% [44]. After the drug molecules’ entrapment, the beads’ porosity decreased due to the high drug concentration, and arrows indicate interconnectivity among the pores. The pore sizes of the GA and GAQ beads range from 10–100 µm, and the mean pore size was determined to be 56.1 ± 34.1 µm (Figure 2b). The estimated pore size was suitable for better cell growth on this artificial matrix.

FTIR spectra of polymer, drug, and GA and GAQ beads showed the proper interactions between the bead constituents (Figure 2c). In the case of gelatin, spectra peaks were at 3440 cm^−1^, 1641 cm^−1^, 1542 cm^−1^, and 664 cm^−1^ for NH and C–O stretching and bending, respectively. Besides that, CN stretching peaks between 1070 and 1240 cm^−1^, –CH_2_ stretching peak at 1455 cm^−1^, and C–H bending at 1395 cm^−1^ were observed. However, in the case of alginate, spectra peaks at 1032 cm^−1^, 1232 cm^−1^, 1638 cm^−1^, 2185 cm^−1^ and 3452 cm^−1^ indicate the glucuronic (G) and manuronic (M) acid functional groups, respectively. The peaks at 885 cm^−1^ and 817 cm^−1^ showed the β-glycosidic linkages between the G and M units of alginate after cross-linking. Similarly, the quercetin FTIR spectra indicate the presence of all essential functional groups’ peaks as reported in the literature [13,22,23,24]. Here, in the GAQ beads, the disappearance and shifting of peaks in the polymeric and quercetin-loaded alginate–gelatin beads indicates the proper interactions, functionalization, and 100% encapsulation of drug molecules.

### 3.2. In Vitro Anti-Inflammatory, Oxidative, and Drug Release Profile

**Figure 3:**
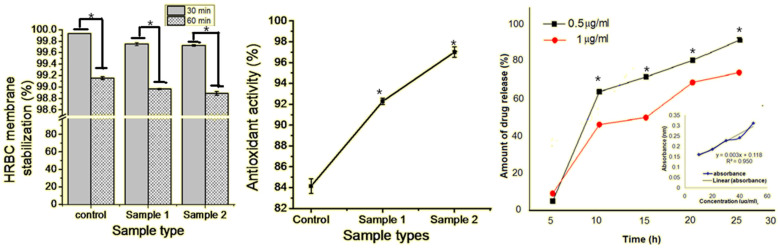
Anti-inflammatory, anti-oxidative, and drug-release profile of the quercetin-loaded gelatin–alginate polymeric beads. Here * indicated the significant difference between the samples at *p* ≥ 0.05.

The polymeric beads showed 100% drug encapsulation on the polymeric matrix without any significant loss of drug (quercetin) occurring in the medium. The drug-loaded beads showed free radical stabilization properties for up to 60 min with no significant inflammatory response occurring for a long time. The anti-oxidative and non-immunogenic nature of all GAQ beads increased by increasing the concentration of quercetin. Subsequent studies showed that the release of quercetin from the surface of the GAQ beads has a cumulative release profile of quercetin (Figure 3). For the calibration curve and release profile study suggested in Samples 1 and 2, from the initial stat to 4 h, approximately 10% of the drug was released to the surrounding medium. However, from 5 h to 10 h, the progressive release of drug molecules occurred―45% of the drug was released from Sample 2, and more than 60% drug was released from Sample 1. The data indicate a burst release and the saturation point after 12 h incubation, with the rapid release of drug molecules with significant anti-oxidative and anti-inflammatory properties (Figure 3). The drug loading efficacy of the GA beads is significantly enhanced with respect to a previously reported quercetin model of different polymer matrices such as poly-caprolactone. ECM has proper interaction up to 50% and good bioactivity [16,38,47].

### 3.3. In Vitro Cell Compatibility

**Figure 4:**
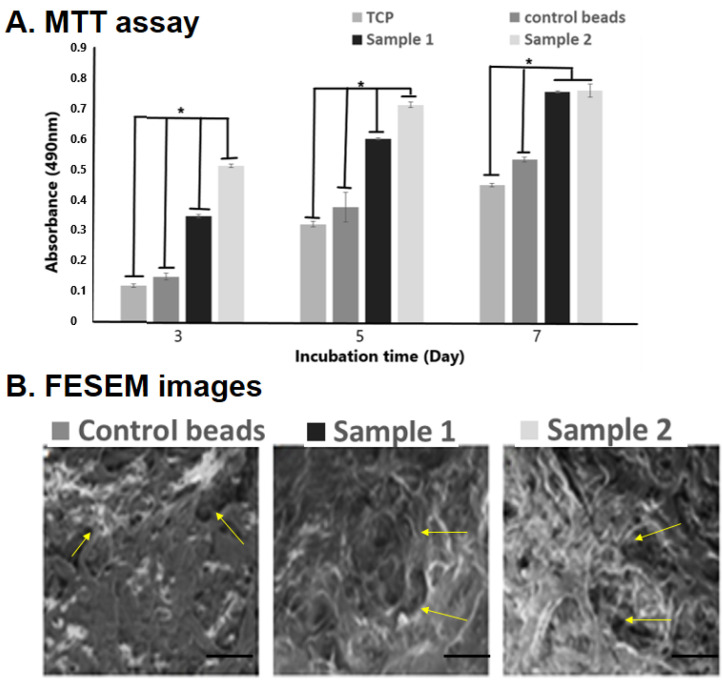
Fabricated polymeric beads’ biocompatibility study: MTT assay to check cell viability (**A**), and cell-seeded matrix FESEM images at 10 kX magnification (100 µm scale) (**B**) to show cell proliferation and adherence over the matrix (yellow arrows). Here * indicated the significant difference between the samples at *p* ≥ 0.05.

The biopolymers of plant–animal origin are biocompatible and non-toxic sources widely used for food and clinical applications. Gelatin and alginate are dressing materials for wound healing, encapsulation, and control of the biological system’s release of biomolecules or drugs [38,39,48]. Here, the MTT assay result showed that cells cultured on the bio-polymeric beads have good cell viability, proliferation, and growth with progression over time (Figure 4). The absorbance of the GAQ beads represented the cell growth, which constantly increased after the incorporation of the quercetin molecule, indicating their biocompatibility, anti-inflammatory, and anti-oxidative effects. Previous studies reported that quercetin has a significant role in cell viability and control of cell division [49,50].

## 4. Conclusions

Quercetin-loaded scaffolds using natural biogenic polymers and bioactive compounds were successfully synthesized without toxic cross-linking agents and surfactants. However, the previous studies on the foaming scaffold methodology were based on using toxic surfactants to stabilize the foam. Here, the natural polymer used for scaffold fabrication has structural resemblances with native tissue ECM for better cell growth and tissue regeneration. The quercetin-loaded beads (GAQ) act as an ideal scaffold enriched with natural flavonoid molecules, having potential anti-inflammatory, anti-oxidative and non-toxicity effects for better cell growth and protecting them from damage. Besides that, the concentration effect of quercetin loaded on the alginate–gelatin polymeric beads showed a significant difference to their biological activity. Therefore, based on the in vitro studies of the successful entrapment and controlled release of quercetin molecules through GA, the modified GAQ beads were evaluated as a novel drug delivery system that could be used to treat several acute to chronic infections with healthy tissue regeneration at the damaged site.

## Figures and Tables

**Table 1:** Chemical composition of drug-loaded scaffolds with different concentrations.

Chemical Components	Control	Sample 1	Sample 2
Gelatin (2%):Alginate (5%)	1:1	1:1	1:1
CaCl_2_ + Quercetin (µg/mL)	10	10:0.5	10:1

**Table 2:** Beadssize, pore-size and porosity.

Beads Types	Control	Sample 1	Sample 2
Bead size (mm)	0.7 ± 0.04	0.67 ± 0.02	0.81 ± 0.01
Porosity%	92 ± 15.10	77 ± 26.81	64 ± 15.65
Pore size (µm)	81 ± 5.27	70 ± 5.27	63 ± 11.27

## Data Availability

Not applicable.
